# Walking intervention and dietary guidance efficacy for overweight people with schizophrenia: An open‐label 12‐week study

**DOI:** 10.1002/pcn5.52

**Published:** 2022-10-25

**Authors:** Taro Kishi, Makoto Okuya, Kenji Sakuma, Yohei Otaka, Eiichi Saitoh, Nakao Iwata

**Affiliations:** ^1^ Department of Psychiatry Fujita Health University School of Medicine Toyoake Aichi Japan; ^2^ Department of Rehabilitation Medicine I Fujita Health University School of Medicine Toyoake Aichi Japan

Individuals with schizophrenia have approximately 10‐year shorter life expectancy compared with the general population.^1^, ^2^ About 60% of the excess mortality is due to physical comorbidities, predominantly cardiovascular diseases (CVD).^3^ A major CVD risk factor is metabolic syndrome, such as being overweight, which is notably higher in schizophrenia patients.^1^, ^2^ Overweight patient risk factors include poorer lifestyle behaviors, reduced access to physical care, frequent comorbid illnesses, and antipsychotic medications.^1^, ^2^ Our cross‐sectional study demonstrated that people with schizophrenia had a significantly higher body mass index (BMI), waist–hip ratio (WHR), body fat mass (BFM), and percent body fat (PBF) compared with healthy controls.^4^

The Japanese Society of Psychiatry and Neurology (JSPN) released “Guide to Preventing Obesity and Diabetes Mellitus Complicated by Schizophrenia” in 2020.^5^ The guide recommends exercise and dietary therapy for overweight people with schizophrenia. A 12‐week open‐label study was therefore conducted to investigate the benefits of walking intervention and dietary guidance for overweight people with schizophrenia.

The study was conducted from February 2021 to March 2022 at Fujita Health University Hospital (UMIN000048435). Both female and male outpatients (20–64 years) with clinically stable schizophrenia were recruited. The *Diagnostic and Statistical Manual of Mental Disorders*, Fifth Edition^6^ was used for diagnostic criteria. Other inclusion criteria were as follows: patients determined to be able to walk 10,000 steps/day^7^ at least 5 days/week using a pedometer and patients with a BMI ≥25. The exclusion criteria were pregnancy or breastfeeding; severe physical illness, such as severe hypertension (blood pressure ≥180/100 mmHg), diabetes mellitus, and neurological or musculoskeletal diseases; and anyone deemed inappropriate to participate by the attending physician. The clinical trial was explained to all the participants who provided written informed consent, and approved by the Fujita Health University Ethics Committee (HM20‐413).

Participants were encouraged to walk at least 10,000 steps/day and were briefed on the dietary guidance in the guide at baseline.^5^ Psychotropic drugs were maintained at the same dose throughout the study. The InBody470 (http://inbody.com/eng/main/Main.aspx) was used to measure BMI, WHR, BFM, PBF, soft lean mass, fat free mass, skeletal muscle mass, skeletal muscle index, and basal metabolic rate at baseline, 4, 8, and 12 weeks. The Clinical Global Impression–Severity (CGI‐S) was used to observe the participants' mental state.^8^ All efficacy measures analyses were based on the observed case population. The Wilcoxon signed rank test was used to evaluate statistically significant differences in each body composition score and CGI‐S score between baseline and endpoint given the small sample size. We performed two subgroup analyses to examine the mean steps/day's impact on the results using the Wilcoxon rank–sum test as follows: (1) subgroup including participants who walked a mean ≥6000 steps/day during the study versus subgroup including participants who walked a mean <6000 steps/day during the study, and (2) subgroup including participants who walked a mean ≥8000 steps/day during the study versus subgroup including participants who walked a mean <8000 steps/day during study. The JMP software (JMP 14.2.0; SAS Institute Inc.) was used to perform all statistics.

The patients' dispositions are shown in Supporting Information: Figure S1. A total of 20 patients were included for baseline analysis. The female proportion was 55.00% and the mean age was 44.70 ± 10.13 years. Daily steps mean number from baseline to 4 weeks, baseline to 8 weeks, and baseline to 12 weeks were 5346.24 ± 3121.18, 5586.07 ± 3120.97, and 5460.91 ± 3365.87, respectively.

Although diastolic blood pressure significantly decreased from baseline to 12 weeks, other outcomes' data were not significantly changed from baseline at each endpoint (Table S1). However, the subgroup including participants who walked a mean ≥8000 steps/day showed decreased systolic blood pressure, body weight, BMI, WHR, BFM, and PBF values compared with the subgroup including participants who walked a mean <8000 steps/day (Table 1). There were no significant differences in all outcomes other than WHR between two subgroups when the average daily steps number was divided into thresholds at 6000 steps (Table 1). For all subgroups, these results might be a Type II error due to the small sample size, although data of most outcomes were not significantly changed from baseline to each endpoint (Table 1). No participant had any adverse events.

**Table 1:** Subgroup analysis results divided by mean daily steps

	≥6000 steps/day vs. <6000 steps/day					≥8000 steps/day vs. <8000 steps/day				
	Subgroup including participants who walked ≥mean 6000 steps/day (*n* = 6)	*p*‐Value of Wilcoxon signed rank test (compared with baseline)	Subgroup including participants who walked <mean 6000 steps/day (*n* = 7)	*p*‐Value of Wilcoxon signed rank test (compared with baseline)	*p*‐Value for subgroup difference	Subgroup including participants who walked ≥mean 8000 steps/day (*n* = 4)	*p*‐Value of Wilcoxon signed rank test (compared with baseline)	Subgroup including participants who walked <mean 8000 steps/day (*n* = 9)	*p*‐Value of Wilcoxon signed rank test (compared with baseline)	*p*‐Value for subgroup difference
CGI‐S	−0.33 ± 0.82	1.0000	−0.14 ± 0.38	1.0000	0.8203	0.00 ± 0.00	1.0000	−0.33 ± 0.71	0.5000	0.3264
Systolic blood pressure (mmHg)	−12.67 ± 8.16	**0.0313**	1.00 ± 14.61	0.8438	0.1161	−16.50 ± 7.19	0.1250	−0.33 ± 12.94	0.9999	**0.0308**
Diastolic blood pressure (mmHg)	−8.67 ± 8.24	0.0938	−4.00 ± 10.72	0.4063	0.4314	−10.00 ± 9.76	0.1250	−4.44 ± 9.54	0.2109	0.2794
Pulse (beats/min)	−3.83 ± 17.85	0.4375	10.43 ± 11.96	0.1250	0.1156	3.25 ± 16.62	1.0000	4.11 ± 16.87	0.5781	0.8772
Body weight (kg)	−2.25 ± 2.64	0.1563	−0.04 ± 1.84	0.8438	0.1531	−3.90 ± 0.55	0.1250	0.20 ± 1.72	0.5156	**0.0087**
Body mass index	−0.90 ± 1.05	0.1250	−0.04 ± 0.69	0.7969	0.1973	−1.55 ± 0.29	0.1250	0.06 ± 0.64	0.4844	**0.0133**
Waist–hip ratio	−0.03 ± 0.03	0.1250	0.01 ± 0.03	0.3750	**0.0411**	−0.05 ± 0.02	0.1250	0.01 ± 0.03	0.2656	**0.0074**
Body fat mass	−1.80 ± 2.62	0.1563	0.76 ± 2.25	0.3750	0.0865	−3.18 ± 1.64	0.1250	0.80 ± 2.07	0.3008	**0.0136**
Percent body fat	−1.25 ± 2.69	0.3438	0.96 ± 2.04	0.3281	0.1161	−2.40 ± 2.28	0.2500	0.98 ± 1.93	0.2617	**0.0449**
Fat‐free mass	−0.45 ± 1.54	0.6250	−0.80 ± 1.53	0.2188	0.5197	−0.73 ± 1.87	0.6250	−0.60 ± 1.41	0.2500	0.9384
Soft lean mass	−0.42 ± 1.47	0.6250	−0.81 ± 1.43	0.2188	0.5192	−0.70 ± 1.78	0.6250	−0.60 ± 1.33	0.2383	0.9383
Skeletal muscle mass	−0.23 ± 0.93	0.6250	−0.42 ± 0.83	0.2031	0.5197	−0.40 ± 1.12	0.6250	−0.31 ± 0.77	0.3008	0.8167
Skeletal muscle index	0.02 ± 0.17	0.9063	−0.21 ± 0.29	0.1563	0.0734	−0.08 ± 0.13	0.5000	−0.12 ± 0.31	0.3906	0.6425
Basal metabolic rate	−10.00 ± 33.85	0.6250	−17.57 ± 32.38	0.2188	0.6166	−16.00 ± 41.04	0.6250	−13.22 ± 29.77	0.2500	0.8167

*Note*: Values in bold indicate statistically significant results. CGI‐S, Clinical Global Impression–Severity.

This study's results suggested that walking at least 8000 steps/day and dietary guidance recommended by the JSPN would likely help patients decrease body weight and body fat. These treatments may be routinely suggested to patients in the clinical setting because they are convenient. However, this study's main limitation was the small sample size. Moreover, the study duration was short. Furthermore, this study did not have a control. Other limitations are shown in Supporting Information. A randomized, controlled trial on these interventions for overweight individuals with schizophrenia is required to obtain more robust evidence.

## AUTHOR CONTRIBUTIONS

Taro Kishi contributed to the conception and study design. All authors contributed substantially to the analysis and interpretation of clinical data. Taro Kishi wrote the first draft of the article. All authors have contributed to and approved the final version of the manuscript. Nakao Iwata, Yohei Otaka, and Eiichi Saitoh supervised this study.

## CONFLICT OF INTEREST

The authors declare no conflict of interest.

## ETHICS APPROVAL STATEMENT

The study was approved by the Ethics Committee of Fujita Health University (HM20‐413).

## PATIENT CONSENT STATEMENT

The clinical trial was described in detail to the subjects, all of whom provided written informed consent.

## CLINICAL TRIAL REGISTRATION

This study was registered in the UMIN Clinical Trials Registry as UMIN000048435.

## Supporting information

Supporting information.

## Data Availability

The entirety of the patients' data cannot be made publicly available as data sharing was not included in the consent.
