# Supplementary material for: Walking intervention and dietary guidance efficacy for overweight people with schizophrenia: An open‐label 12‐week study
Source: PCN Rep. 2022 Oct 25;1(4):e52. doi: 10.1002/pcn5.52 (PMC11114403; doi:10.1002/pcn5.52)
Supplement: Supplementary file 1 — Supporting information. [file PCN5-1-e52-s001.docx]

**Figure S1. Participants disposition**


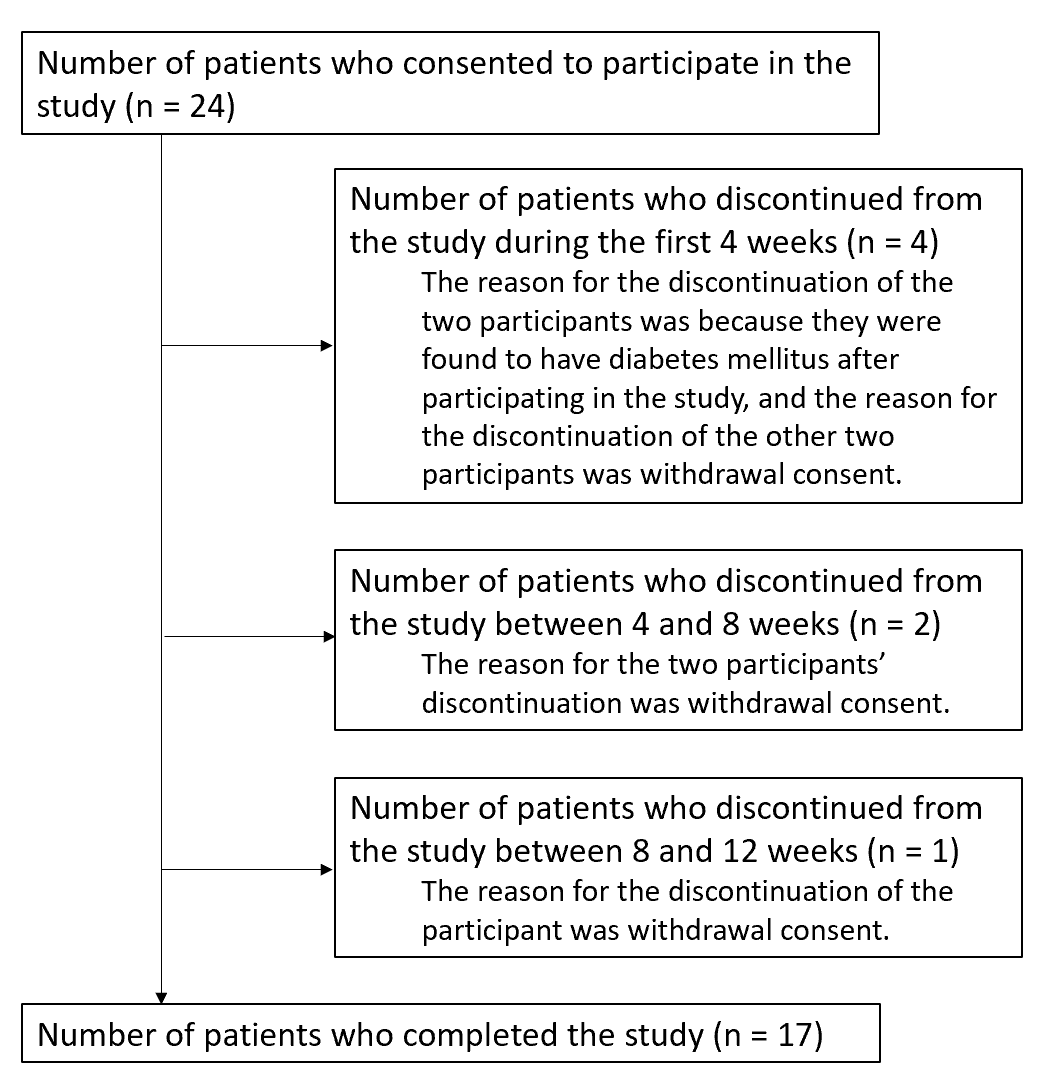


**Table S1. Change values in all outcomes from baseline to each observational visit**

|  | Baseline (n = 20) | 4 weeks (n = 20) | | 8 weeks (n = 18) | | 12 weeks (n = 17) | |
| --- | --- | --- | --- | --- | --- | --- | --- |
|  | Mean ± SD | Change mean value ± SD from baseline to 4 weeks | P-value of Wilcoxon signed rank test (compared with baseline) | Change mean value ± SD from baseline to 8 weeks | P-value of Wilcoxon signed rank test (compared with baseline) | Change mean value ± SD from baseline to 12 weeks | P-value of Wilcoxon signed rank test (compared with baseline) |
| CGI-S | 3.40 ± 0.60 | -0.15 ± 0.59 | 0.5000 | -0.22 ± 0.55 | 0.2500 | -0.18 ± 0.53 | 0.5000 |
| Systolic blood pressure (mmHg) | 136.60 ± 21.79 | -2.55 ± 12.43 | 0.3633 | -2.67 ± 14.68 | 0.6024 | -4.18 ± 15.02 | 0.2362 |
| Diastolic blood pressure (mmHg) | 82.45 ± 12.08 | -0.80 ± 12.00 | 0.1571 | -2.83 ± 15.43 | 0.3840 | -6.71 ± 9.27 | **0.0107** |
| Pulse (beats/min) | 91.90 ± 13.25 | -1.80 ± 15.61 | 0.2506 | -4.17 ± 10.12 | 0.0753 | 1.76 ± 16.34 | 0.7299 |
| Body weight | 82.98 ± 25.29 | -0.15 ± 1.28 | 0.7568 | -0.78 ± 1.71 | 0.0794 | -0.78 ± 2.43 | 0.3347 |
| Body mass index | 31.27 ± 7.57 | -0.08 ± 0.49 | 0.5579 | -0.33 ± 0.15 | 0.0643 | -0.33 ± 0.93 | 0.3349 |
| Waist-hip ratio | 0.93 ± 0.06 | -0.01 ± 0.02 | 0.1624 | -0.01 ± 0.03 | 0.3047 | -0.01 ± 0.03 | 0.2979 |
| Body fat mass | 33.52 ± 15.70 | -0.22 ± 1.25 | 0.2419 | -0.37 ± 2.00 | 0.4799 | -0.35 ± 2.67 | 0.6524 |
| Percent body fat | 39.53 ± 7.26 | -0.16 ± 1.30 | 0.4167 | -0.18 ± 2.18 | 0.7904 | -0.14 ± 2.51 | 0.8452 |
| Fat free mass | 49.46 ± 12.60 | 0.07 ± 1.27 | 0.7301 | -0.39 ± 1.54 | 0.1326 | -0.42 ± 1.42 | 0.2479 |
| Soft lean mass | 46.70 ± 12.00 | 0.08 ± 1.23 | 0.7603 | -0.37 ± 1.41 | 0.1506 | -0.42 ± 1.34 | 0.2564 |
| Skeletal muscle mass | 27.39 ± 7.50 | 0.04 ± 0.74 | 0.7896 | -0.23 ± 0.85 | 0.1057 | -0.23 ± 0.78 | 0.2564 |
| Skeletal muscle index | 7.85 ± 1.54 | 0.01 ± 0.21 | 1.0000 | -0.06 ± 0.24 | 0.4291 | -0.04 ± 0.26 | 0.7500 |
| Basal metabolic rate | 1438.40 ± 272.04 | 1.53 ± 27.48 | 0.7604 | -8.89 ± 32.20 | 0.1444 | -9.35 ± 30.47 | 0.2684 |

Values in bold indicate statistically significant results.

**Supplementary text**

Our study had the following limitations.

First, we did not know whether participants were originally obese or antipsychotic-induced weight gain. Second, the reasons for poor walking adherence of some participants might be associated with severity of psychopathology (especially negative symptoms) and the presence of extrapyramidal symptoms. However, we did not evaluate the psychopathology and the extrapyramidal symptoms of the participants.
